# Evidence of epigenetic landscape shifts in mucopolysaccharidosis IIIB and IVA

**DOI:** 10.1038/s41598-024-54626-4

**Published:** 2024-02-17

**Authors:** Viviana Vargas-López, Luisa F. Prada, Carlos J. Alméciga-Díaz

**Affiliations:** Institute for the Study of Inborn Errors of Metabolism, Faculty of Science, Pontificia Universidad Javeriana, Cra. 7 No. 43-82 Edificio 54, Laboratorio 305A, Bogotá D.C., 110231 Colombia

**Keywords:** Cell biology, Molecular biology, Molecular medicine

## Abstract

Lysosomal storage diseases (LSDs) are a group of monogenic diseases characterized by mutations in genes coding for proteins associated with the lysosomal function. Despite the monogenic nature, LSDs patients exhibit variable and heterogeneous clinical manifestations, prompting investigations into epigenetic factors underlying this phenotypic diversity. In this study, we focused on the potential role of epigenetic mechanisms in the pathogenesis of mucopolysaccharidosis IIIB (MPS IIIB) and mucopolysaccharidosis IVA (MPS IVA). We analyzed DNA methylation (5mC) and histone modifications (H3K14 acetylation and H3K9 trimethylation) in MPS IIIB and MPS IVA patients’ fibroblasts and healthy controls. The findings revealed that global DNA hypomethylation is present in cell lines for both diseases. At the same time, histone acetylation was increased in MPS IIIB and MPS IVA cells in a donor-dependent way, further indicating a shift towards relaxed open chromatin in these MPS. Finally, the constitutive heterochromatin marker, histone H3K9 trimethylation, only showed reduced clustering in MPS IIIB cells, suggesting limited alterations in heterochromatin organization. These findings collectively emphasize the significance of epigenetic mechanisms in modulating the phenotypic variations observed in LSDs. While global DNA hypomethylation could contribute to the MPS pathogenesis, the study also highlights individual-specific epigenetic responses that might contribute to phenotypic heterogeneity. Further research into the specific genes and pathways affected by these epigenetic changes could provide insights into potential therapeutic interventions for these MPS and other LSDs.

## Introduction

Monogenic diseases are characterized by Mendelian inheritance of mutations in single genes. Despite this, patients with monogenic diseases often show wide variability between symptoms, which has led to research^1,2^. Epigenetic mechanisms, including DNA methylation, histone modifications, and microRNAs, have been proposed to regulate the phenotypic variation observed in patients with these disorders^2^. Among monogenic diseases, lysosomal storage disorders (LSDs), a group of inherited metabolic diseases, represent a significant subset where epigenetics might contribute to the variable clinical presentations observed at different stages of the disease^2,3^.

Despite the potential role of epigenetics on LSDs phenotype variability, only limited research has been published on this topic. Specifically, DNA methylation, the addition of a methyl group to a cytosine (5mC) frequently occurring at CpG dinucleotides in gene promoters to silence genes, has received the most attention in this context. Methylation also plays a critical role in human genetic diseases through point mutations due to the deamination of 5-methylcytosine present within CpG dinucleotides^4^. It has been reported that methylation levels are increased in patients with mucopolysaccharidosis type II (MPS II, Hunter syndrome) and IVA (MPS IVA, Morquio A syndrome) compared with healthy controls^5,6^. In this regard, a relationship was reported between mutations and hypermethylation in exon 2 to intron 3 of the iduronate-2-sulfatase (IDS) gene for MPS II patients^6^. Also, a study on methylation changes in the N-acetylgalactosamine-6-sulfate sulfatase (*GALNS*) gene involved in MPS IVA showed that transitional mutations that originated after deamination at the CpG dinucleotides were found in 29% of the point mutations in MPS IVA patients, suggesting that methylation in these CpG regions increase the probability of gene mutation^5^. Dysregulated DNA methylation in CpG sites throughout the genome and perturbed methionine cycle have also been observed in Fabry patients' endothelial cells^7^.

Interestingly, metabolic changes have been involved in epigenetic regulation, specifically methylation and acetylation, and represent new forms of disease pathogenesis^8^. Changes in methylation patterns could be associated with further metabolic dysregulation. For example, a study in the cerebellum of a Niemann-Pick Type C disease mouse model found decreased expression of methylation proteins, DNA methyltransferases, and methyl-CpG binding proteins^9^. At the same time, a dysregulation in amino acid metabolism suggested disturbances in folate and methylation pathways^9^.

Histone modifications and microRNAs have been less explored in the context of LSDs. It has been reported that histone H4 hypoacetylation is associated with reduced expression of neurotrophic factors in patients with Gaucher disease type I^10^. Also, miRNAs screenings in LSDs, including Niemann-Pick type C, Gaucher disease, and multiple sulfatase deficiency, have identified several changes in the miRNAs' levels targeting specific genes for these diseases and genes of different metabolic pathways. These results suggest that miRNAs have potential significance as modulators of pathways associated with lysosomal functions^11^.

Furthermore, recent transcriptomic and proteomic studies have revealed significant transcriptional and translational changes in fibroblasts from patients with different MPS^12–15^ and a MPS VII^16^. Also, a system biology study of MPS showed that impairment in glycosaminoglycan-related enzymes may induce several metabolic changes, including cellular respiration, mitochondrial process, amino acid and lipid metabolism, and ion exchange, among others^17^. Taken together, this evidence suggests that gene variants, in genes associated with LSDs, induce different molecular and cellular changes, among which epigenetics may play an essential role.

Among MPSs, MPS III and MPS IV are the only ones categorized into distinct subtypes given their distinct disease progression patterns despite similar GAG accumulation^18^. This feature makes them particularly intriguing for exploring the role of epigenetics in LSDs. MPS IVA, caused by gene variants in the *GALNS* gene leads to the storage of chondroitin-6-sulfate and keratan sulfate, resulting in severe skeletal abnormalities that affect bones, joints, and ligaments, as well as lung and heart disease in patients^19^. Conversely, MPS IIIB arises from gene variants in the N-acetylglucosaminidase (*NAGLU*) gene that leads mainly to neurodegeneration due to the lysosomal storage of heparan sulfate. In Colombia, MPS IVA stands as the most prevalent MPS subtype, with a combined prevalence of 1.98 and approximately 200 diagnosed patients^20–22^, while MPS IIIB ranks as the third most common, with 10.8% of the diagnosed patients and an estimated incidence of 0.36 affected individuals per 100,000 live births^22^. During the last few years, we have studied the cellular and molecular mechanisms of MPS IVA and MPS IIIB^17,23,24^, testing the potential of different therapeutic alternatives including recombinant enzymes^25–29^, gene therapy^30–34^, and pharmacological chaperones^35–37^. Given the profound impact of MPS IIIB and IVA both within Colombia and globally, it is crucial to enhance our understanding of their molecular basis enabling expedited and effective diagnostic and therapeutic interventions.

Here, we evaluated the amount and spatial distribution of epigenetic marks shifts related to gene silencing (5mC), constitutive heterochromatin (histone H3 lysine 9 trimethylation—H3K9me3), and open chromatin (histone H3 lysine 14 acetylation—H3K14ac), in skin fibroblasts from MPS IIIB and MPS IVA, to shed light on the epigenetic landscape shifts in these disorders. This research may contribute to our understanding of the underlying molecular mechanisms driving these disorders, potentially paving the way for novel therapeutic interventions and personalized treatment approaches.

## Materials and methods

### Cells

Human fibroblasts (wild type: GM00613; MPS IIIB: GM02931/GM01426: MPS IVA: GM01259/GM00958) were obtained from the Coriell Institute for Medical Research. Details on cell lines are presented in Table 1. Fibroblasts were maintained in DMEM plus 15% FBS, 100 U/mL penicillin, and 100 µg/mL streptomycin (Gibco, USA). Cells were incubated at 37 °C in a humidified atmosphere containing 5% CO_2_. Cells between 10 and 18 passages were used in all experiments. The selection of these specific cell lines was based on discernible factors such as documented mutations, enzymatic activity, and our team's extensive prior experience with them.

**Table 1:** Cell lines information.

Cell line	Disease	Protein	Mutation	Age (y/o)	Sex
GM00613	Unaffected	N/A	N/A	Unknown	Female
GM02931	MPS IIIB	NAGLU	P358L	3	Female
GM01423	MPS IIIB	NAGLU	E153K	1	Female
GM01259	MPS IVA	GALNS	R94C/A393S	14	Female
GM00958	MPS IVA	GALNS	A393S	12	Male

### Real-time quantitative PCR

Total RNA was isolated using the Monarch® Total RNA Miniprep Kit (New England Biolabs; USA). RNA integrity was checked in a 2% agarose denaturing gel and quantified using a NanoDrop 1000 Spectrophotometer (Thermo Fisher Scientific, USA). 0.5 µg of total RNA was reverse transcribed using a High-Capacity cDNA Reverse Transcription Kit (Applied Biosystem; USA). For each sample, five reactions were run. Each reaction contained 100 ng of cDNA, forward and reverse primers at 0.1 µM final concentration (primer sequences are presented in Table 2), 10 µL of 2X master mix, and 0.4 µl ROX reference dye (DyNAmo HS SYBR Green qPCR Kit, Thermo Scientific #F-410L). qPCR reactions were performed in a QuantStudio3 Real-Time PCR system (Applied Biosystems; Thermo Fisher Scientific, Inc) using the recommended protocol (initial incubation at 50 °C for 2 min, polymerase activation 95 °C for 15 min, 40 cycles of amplification at 95 °C for 10 s/60 °C for 60 s. The melting curve for each pair of primers was also performed after amplification (95 °C–15 s, 60 °C–60 s, 95 °C–1 s). Relative gene expression was calculated using the 2^–ΔΔCq^ method. Results were expressed as the ratios of target genes against the housekeeping gene ß-actin and expression in wild-type samples.

**Table 2:** List of primers used for qPCR.

Gene Name	Primer Sequence 5’–3’
*NAGLU*	Forward: CAGAAGGAAGGAGCAGGAGT
	Reverse: ATGTTCCCGAGGCTGTCAC
*GALNS*	Forward: ACAGGGCCATTGATGGCCTCAACCTCCT
	Reverse: GCTTCGTGTGGTCTTCCAGATTGTGAGTTG
*β-ACTIN*	Forward: GGACATCCGCAAAGACCTGTA
	Reverse: GCTCAGGAGGAGCAATGATCT

### Enzyme activity assays

For enzyme activity assays, cells were harvested and lysed in 1% deoxycholate and 1 mM phenylmethylsulfonyl fluoride (PMSF; Sigma). Samples were centrifuged at 14.000 rpm and 4 °C for 10 min. NAGLU activity was tested using the substrate 4-methylumbelliferyl-2-acetamide-2-deoxy-deoxy-alpha-D-glucopyranoside at 2 mM concentration (Abcam ab144785, resuspended in 0.2 M sodium acetate pH 4.5 and 0.5% Triton X-100). For each technical replicate, 50 μL of sample and 50 μL of the substrate were added. The reaction was incubated for 1 h at 37 °C. After this, the reaction was stopped by adding 200 μL of 0.17 M glycine-carbonate stop solution pH 9.8 (composition per liter: glycine 12.6 g, Na_2_CO_3_ 18 g). Reactions were performed in 96-well black flat-bottom polystyrene not treated microplates (Corning; USA). Similarly, GALNS activity was measured using 4-methylumbelliferyl-β-d-galactopyranoside-6-sulfate (Toronto Chemicals Research, North York, ON, Canada) at a 2 mM concentration. Briefly, for each technical replicate, 10 µL of the sample was incubated with 20 µL of the substrate for 17 h at 37 °C. Then, 2 µL of β-galactosidase (10 mg/mL; Sigma) was added, and the samples were incubated for another 4 h. Finally, 150 µL of stop buffer (Glycine-carbonate pH: 9.8) was added, and the reaction was placed in 96-well black flat-bottom microplates. Fluorescence was measured using a Berthold Technologies Twinkle LB 970 fluorometer at 350/450 nm excitation/emission. To determine the relative units of fluorescence associated with 1 nmol, a standard curve between 0.0625 and 2 μM in 0.17 M was made using 4-methylumbelliferone (4MU, Sigma; USA). Enzyme activity unit (U) was defined as the amount of enzyme catalyzing the hydrolysis of 1 nmol of substrate per hour. Finally, specific activity was expressed as U mg^−1^ of total protein determined using the BCA Protein Assay Kit (Thermo Fisher Scientific, USA). At least three biological replicates were performed to assess enzyme activity levels.

### Immunofluorescence

Cells were seeded in circular coverslips 1.5H coated with poly-D-lysine in a 24-well plate and maintained for 24 h in standard cell culture conditions until they reached 80% confluency. Then, cells were washed with 1X PBS and fixed with freshly prepared 4% Paraformaldehyde in 1X PBS for 10 min. Cells were washed with 1X PBS 3 times and then permeabilized for 30 min with 0.5% Triton X-100 in 1X PBS. For 5mC immunofluorescence assays, cells were incubated in 1N HCl denaturing solution for 25 min and neutralized in 0.1 M Tris–HCl (pH 8.3) for 10 min. Cells were blocked in a blocking buffer (BSA 3% w/v in PBS-Triton X 0.5%) for 1 h. After blocking, cells were incubated with primary antibodies diluted in blocking buffer for 24 h at 4 °C (mouse anti-5mC Zymo A3002 1:250; rabbit anti-H3K14ac, Abcam ab52946, 1:1000; or rabbit anti-H3K9me3, Abcam ab176916, 1:4000). After incubation cells were washed with 1X PBS 3 times and then incubated 1 h at room temperature with secondary antibodies in blocking buffer (Goat anti-Rabbit IgG (H + L) Cross-Adsorbed Secondary Antibody, Alexa Fluor™ 594, Invitrogen A-11012, 1:500, or Goat anti-Mouse IgG (H + L) Cross-Adsorbed Secondary Antibody, Alexa Fluor™ 488, Invitrogen A-11029, 1:500). Cells were washed in 1X PBS 3 times, and stained with Hoechst (33342-H3570-1:200.000) for 10 min. Finally, cells were washed in 1X PBS 3 times. Coverslips were mounted in slides using ProLong™ Gold Antifade Mountant Green (Thermo Fisher, P36930).

### Confocal imaging

**Figure 1:**
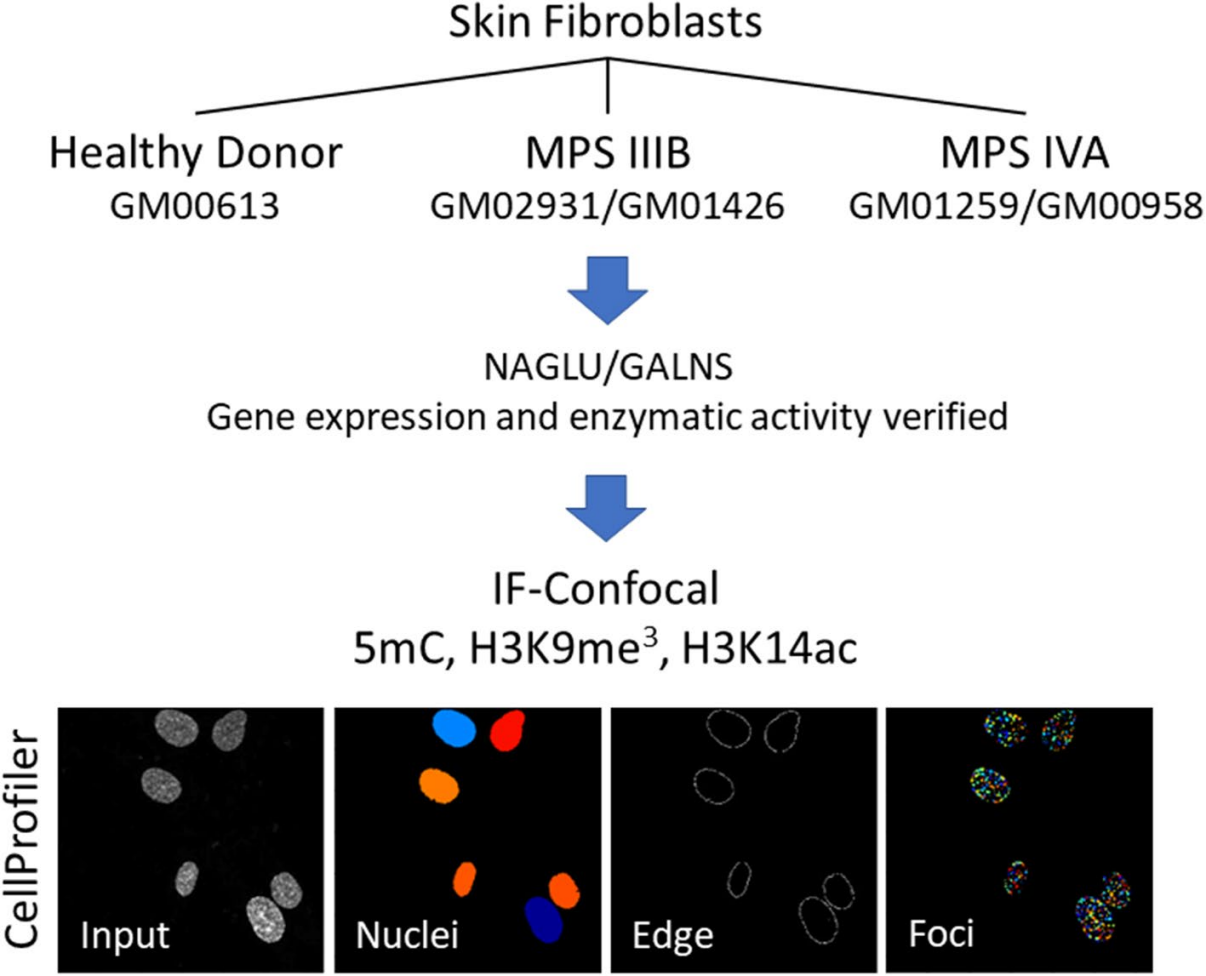
Summary of methodology. Skin fibroblasts from patients with MPS IIB or MPS IVA were compared with cells from a healthy donor to evaluate the potential role of epigenetics. First, gene expression and enzymatic activity for NAGLU and GALNS were tested. Expression levels of epigenetic markings related to gene silencing (5mC), transcriptionally silent heterochromatin (H3K9me3), and transcriptionally active chromatin (H3k14ac) were compared in cell’s nuclei using confocal microscopy and fluorescence intensity and segmentation analysis with CellProfiller.

Cells were imaged using an Olympus FV1000 inverted microscope using 60 × /1.49 NA oil objective and an additional 2X digital zoom. Z-planes were acquired with 0.30 μm intervals using Olympus FV1000 software. 2D Z-stacks projected images on ImageJ were used as input for analysis and quantification. Integrated intensity (representing the relative abundance of the marker), peripheric intensity (representing the relative abundance of the marker at the edge of each nucleus), and foci intensity (representing the intensity of all foci within a cell nucleus calculated by multiplying foci intensity by foci number) fluorescence levels in each cell nucleus were analyzed using a customized CellProfiler pipeline adapted from previously validated pipelines^38,39^. An example of nuclear segmentation, nuclear edges, and identified foci features analyzed with a CellProfiller pipeline is presented in Fig. 1. At least 30 cells per cell line were included for each of at least three immunostaining replicates.

### Statistical analysis

Non-parametric one-way ANOVA analyses were performed to compare wild-type cells versus patient’s cells. Kruskal–Wallis one-way analysis of variance was used for qPCR following Dunn’s multiple comparisons test was used as post hoc analysis. Brown-Forsythe ANOVA test was used for enzymatic activity and epigenetic marking assays using mean and pooled variance of separate biological replicates, Tukey's multiple comparisons test was used as post hoc analyses. All data were analyzed in GraphPad Prism version 8.0.0 for Windows (GraphPad Software, San Diego, California, USA).

## Results

### Enzyme activity and gene expression in MPS IIIB and MPS IVA fibroblasts

**Figure 2:**
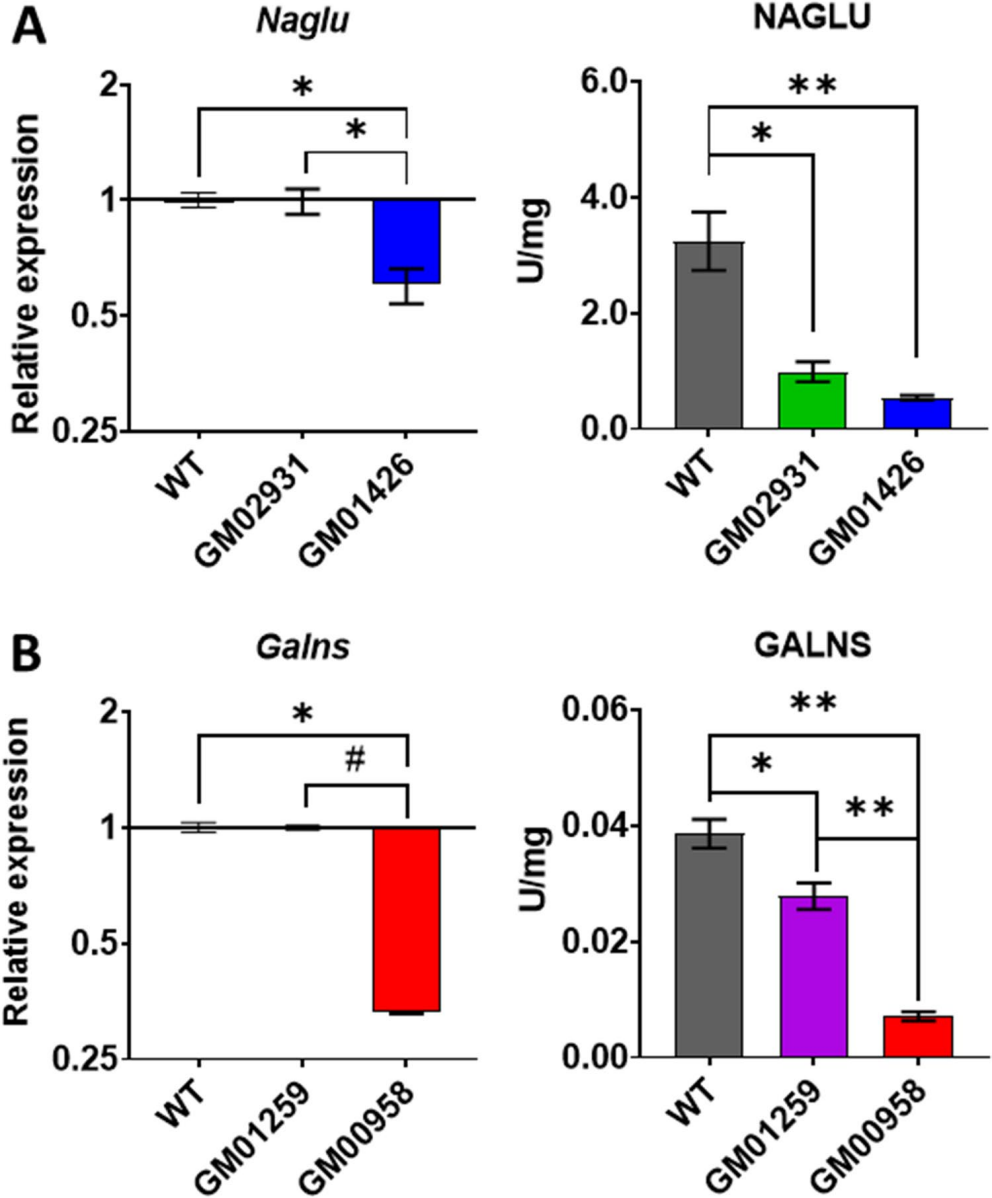
NAGLU (**A**) and GALNS (**B**) gene expression and enzyme activity in healthy and patient’s fibroblasts. Enzymatic activity was further reduced in MPS fibroblasts with the lower mRNA levels. Mean and ± SEM are presented. *p < 0.05, **p < 0.01 compared to wild-type (WT, healthy donor) fibroblasts from a healthy donor. ^#^ < 0.05 between affected cell lines.

At the expression level, it was observed that *NAGLU* mRNA levels (*H*_(2)_ = 9.380, *p* = 0.0029, Fig. 2A-left) were significantly reduced in GM01426 cells (*p* = 0.0216) but not for GM02931 compared to healthy donor fibroblasts. However, NAGLU activity in MPS IIIB fibroblasts (*F*_(2.000, 2.494)_ = 66.26, *p* = 0.0069, Fig. 2A-right) was significantly reduced in both GM02931 (*p* = 0.0191) and GM01426 (*p* = 0.0200), as expected.

In the case of MPS IVA fibroblasts, *GALNS* mRNA levels (*H*_(2)_ = 9.500, *p* = 0.0021, Fig. 2B-left) were significantly reduced in GM00958 cells (*p* = 0.014), but not in GM01259. However, significant reduction in GALNS activity (*F*_(2.000, 4.389)_ = 191.3, *p* < 0,0001, Fig. 2B-right) was observed in both GM01259 (*p* = 0.0117) and GM00958 (*p* = 0.0017) fibroblasts. In addition, GM00958 cells had lower GALNS activity than GM01259 cells (*p* = 0.0058).

### 5mC, a DNA methylation marker, is reduced in MPS IIIB cells

**Figure 3:**
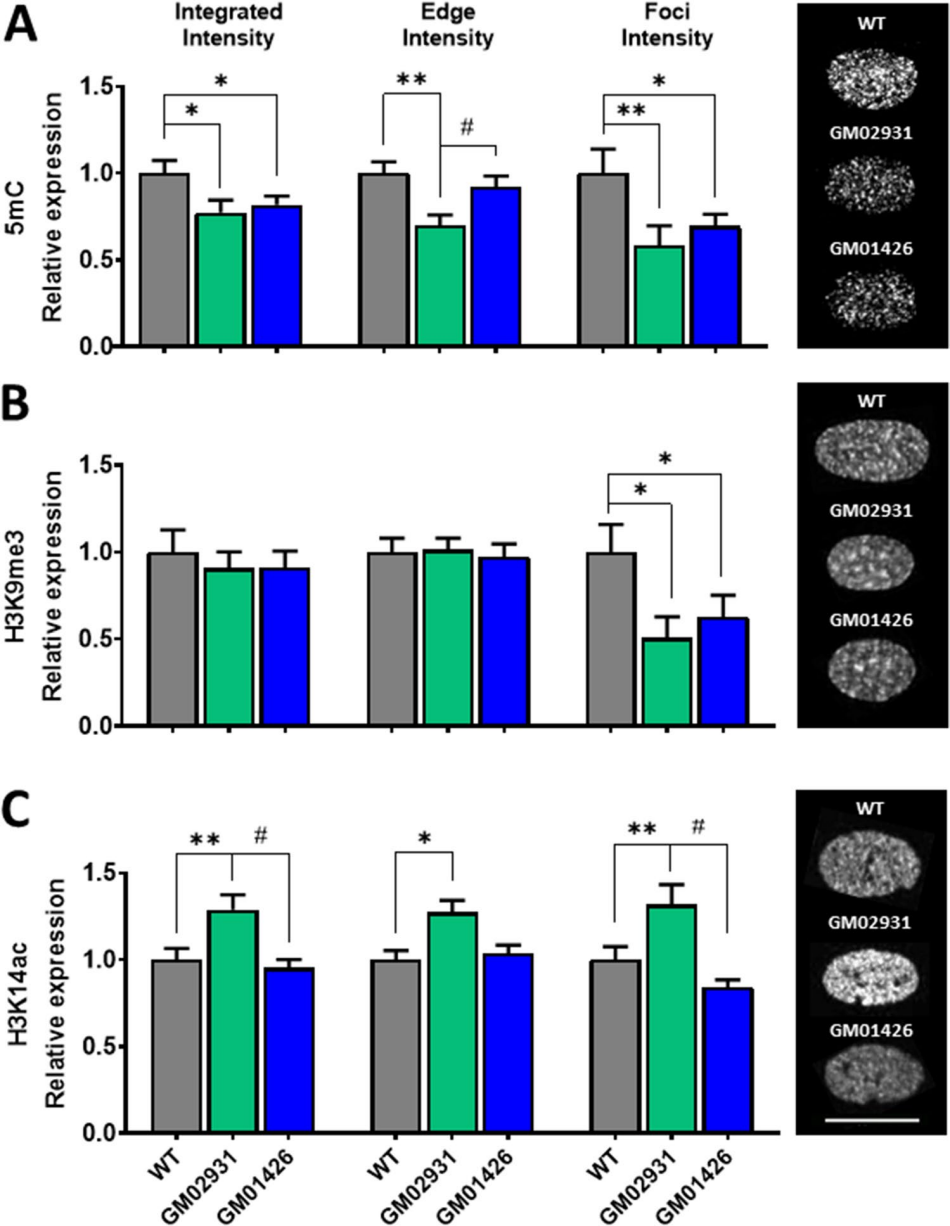
Evaluation of epigenetic markers in MPS IIIB skin fibroblasts. (**A**) Gene methylation marker (5mC) shows a global (left) peripheral (center) and clustering (right) reduction in both MPS fibroblasts. (**B**) Constitutive heterochromatin marker (H3K9me3) shows a clustering (right) reduction in both MPS fibroblasts. (**C**) Euchromatin marker (acH3K14) shows a global (left) peripheral (center) and clustering (right) reduction only in GM02931 but not in GM01426 fibroblasts. Bars represent mean and pooled variance. *p < 0.05, **p < 0.01 as compared to WT. ^#^ < 0.05 as compared to affected cell lines. Representative images are presented on the right of the figure. The white line in the bottom-right represents 20 µm.

5-Methylcytosine (5mC) is a DNA modification involving the addition of a methyl group to the carbon 5 position of the cytosine (C) ring. In humans, DNA methylation mainly occurs at CpG sites, commonly found near gene promoters, but can also be found in other genomic regions, such as repetitive DNA sequences and intergenic regions^40^. Generally, higher levels of 5mC are associated with gene repression or reduced gene expression, but also maintenance of chromosomal integrity and regulation of DNA recombination in mammals^41^. Here, we evaluated the changes in the total 5mC levels (integrated intensity) and its distribution pattern in the nucleus (peripheral and total foci intensity). Specifically, integrated intensity (*F*_(2,6)_ = 10.04, *p* = 0.0122, Fig. 3A-left) levels were significantly reduced in both GM02931 (*p* = 0.0130) and GM01426 (*p* = 0.0335) MPS IIIB fibroblasts. Similarly, peripheral intensity (*F*_(2,6)_ = 18.92, *p* = 0.00262, Fig. 3A-center) levels were reduced in GM02931 (*p* = 0,0025), while foci intensity levels (*F*_(2,6)_ = 11.29, *p* = 0.0092, Fig. 3A-right) were also reduced in both GM02931 (*p* = 0.0089) and GM01426 (*p* = 0.0352). Overall, these results suggest a DNA hypomethylation in MPS IIIB fibroblasts, which may indicate a potential de-repression of normally silenced genes that could lead to important aspects related to the pathophysiology of the disease.

### H3K9me^3^, a transcriptionally silent heterochromatin marker, displays cluster signal reduction in MPS IIIB cells

Histone H3 Lysine 9 trimethylation (H3K9me^3^) is a histone modification recognized as an epigenetic hallmark involved in gene silencing and constitutive heterochromatin in repetitive DNA sequences, centromeres, and telomeres^42–44^. In healthy mature cells, H3K9me^3^ is distributed in isolated islands and foci, mainly located at the nuclear periphery and nucleoli^42–44^. Our results showed that total (*F*_(2,6)_ = 0.7467, *p* = 0.5133, Fig. 3B-left) and peripheral (*F*_(2,6)_ = 0.2099, *p* = 0.08164, Fig. 3B-center) H3K9me^3^ levels were not altered in MPS IIIB fibroblasts. However, H3K9me^3^ foci (*F*_(2,6)_ = 10.70, *p* = 0.0105, Fig. 3B-right) were reduced in both GM02931 (*p* = 0.0105) and GM01426 (*p* = 0.0347) fibroblasts, a change congruent with the observed global reduction in DNA methylation in MPS IIIB fibroblasts. These results suggest a limited *NAGLU* mutation and HS storage impact in the constitutive heterochromatin. However, the foci signal reduction may be associated with changes in H3K9me^3^ spatial distribution, critical for pericentromeric gene repression and senesce regulation^45,46^, and could be associated with reported DNA oxidation and damage observed in MPS IIIB^47^.

### H3K14ac, a transcriptionally active chromatin marker, is differentially altered in MPS IIIB fibroblasts in a donor-dependent manner

Histone H3 Lysine 14 acetylation (H3K14ac) is a histone marker of euchromatin linked to increased gene expression by recruiting transcription factors for assembling the preinitiation complex of transcriptional machinery^48,49^. In healthy cells, H3K14ac is often enriched in genome regions where genes are actively transcribed, including promoters, enhancers, and other regulatory elements associated with gene activation^50,51^. We observed that total H3K14ac levels (*F*_(2,6)_ = 14.11, *p* = 0.0298, Fig. 3C-left) were increased in GM02931 (*p* = 0.0492) but non-altered in GM01426 (*p* = 0.0776) fibroblasts. Similarly, H3K14ac levels in the periphery (*F*_(2,6)_ = 13.07, *p* = 0.0330, Fig. 3C-center) were higher in GM02931 (*p* = 0.0363) but non-changed GM01426 cells (*p* = 0.8017). H3K14ac foci levels (*F*_(2,6)_ = 16.36, *p* = 0.0243, Fig. 3C-right) were also higher in GM02931 (*p* = 0.0472) compared to healthy donor fibroblasts but remained unchanged in GM01426 (*p* = 0.0282) fibroblasts. These results highlight the potential role of individual differences in histone acetylation in phenotypic differences between MPS IIIB patients. However, these results are also consistent with an imbalance between heterochromatin and euchromatin in favor of euchromatin, at least in some patients. These differences may play a role in the differences in phenotypes observed between MPS IIIB patients.

### Global 5mC signal is reduced in MPS IVA fibroblasts

**Figure 4:**
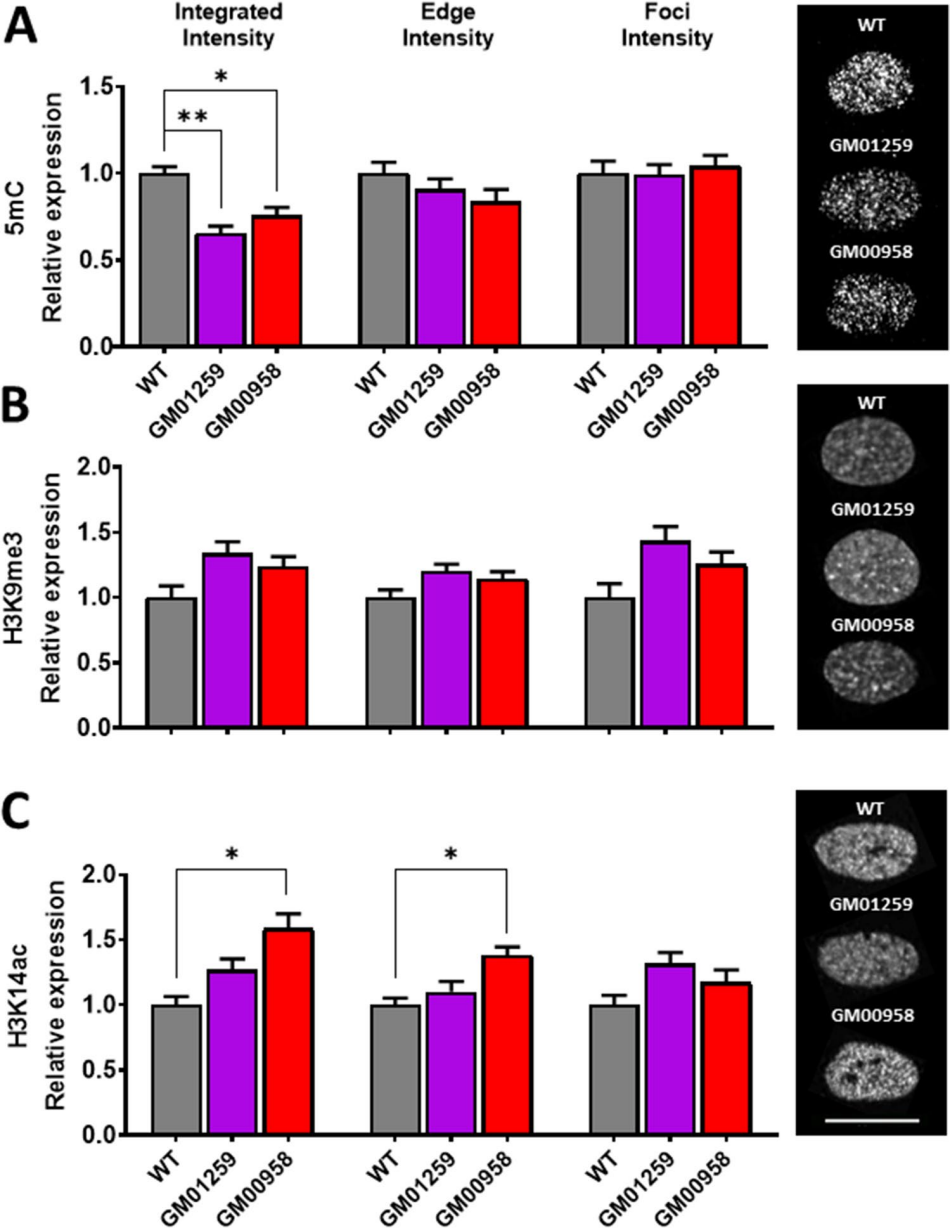
MPS IVA skin fibroblasts exhibit epigenetic changes. (**A**) Gene methylation marker (5mC) shows a global (left) reduction in both MPS cells. (**B**) No changes were observed for the silent heterochromatin marker (H3K9me3). (**C**) Euchromatin marker (acH3K14) shows a global (left) peripheral (center) reduction only in GM02931 but not in GM01426 cells. Bars represent mean and pooled variance. *p < 0.05, **p < 0.01 as compared to WT. Representative images are presented on the right of the figure. The white line in the bottom-right represents 20 µm.

As observed in MPS IIIB fibroblasts, the constitutive heterochromatin marker 5mC was globally reduced in MPS IVA fibroblasts (*F*_(2,6)_ = 34.94, *p* = 0.0084, Fig. 4A-left) as indicated by diminished integrated intensity levels in both, GM01259 (*p* = 0.0080) and GM00958 (*p* = 0.0212) cells. Neither 5mC peripheral levels (*F*_(2,6)_ = 3.256, *p* = 0.1761, Fig. 4A-center) nor clusters signal intensity were altered (*F*_(2,6)_ = 0.3611, *p* = 0.7236, Fig. 4A-right). These findings are consistent with global hypomethylation in MPS IIIB fibroblasts and emphasize the potential role of DNA methylation in the pathophysiology of MPSs.

### H3K9me3 is not altered in MPS IVA fibroblasts

The transcriptionally silent heterochromatin marker H3K9me^3^ was un-altered in MPS IVA fibroblasts as indicated by global (*F*_(2,5)_ = 8.496, *p* = 0.0581, Fig. 4B-left), peripheral (*F*_(2,5)_ = 7.511, *p* = 0.0679, Fig. 4B-center) and cluster levels (*F*_(2,5)_ = 8.552, *p* = 0.0551, Fig. 4B-right). As observed in MPS IIIB fibroblasts, H3K9me^3^ levels were little to non-impaired in MPS IVA, indicating that transcriptionally silent heterochromatin remains mainly intact in these MPS.

### Global and peripheral H3K14ac is differentially affected in MPS IVA cells in a donor-dependent way

Global levels of the transcriptionally active chromatin marker H3K14ac (*F*_(2,6)_ = 22.01, *p* = 0.0161, Fig. 4C-left) were increased in GM00958 (*p* = 0.0143) but not affected in GM01259 fibroblasts (*p* = 0.1124) compared to healthy donor cells. Similarly, H3K14ac peripheral levels (*F*_(2,6)_ = 17.39, *p* = 0.00224, Fig. 4C-center) were increased in GM00958 (*p* = 0.0218) but not affected in GM01259 (*p* = 0.404) fibroblasts. However, cluster signal levels were similar in both MPS IVA fibroblasts compared to those observed in healthy donor cells (*F*_(2,6)_ = 6.814, *p* = 0.0766, Fig. 4C-right). Histone acetylation changes observed in MPS IVA fibroblasts resemble those observed in MPS IIIB where histone hyperacetylation was observed only for one cell line. Potential explanations for these differences must consider specific point mutations and individual genomes for each cell line. These results highlight the potential role of individual differences in epigenetic mechanisms in MPS and support a potential imbalance between heterochromatin and euchromatin in favor of euchromatin in some MPS patients, possibly playing a role in the reported phenotypic heterogeneity between patients.

## Discussion

This study evaluated some epigenetic changes in MPS IIIB and IVA fibroblasts. The levels of DNA methylation (5mC), histone H3 acetylation (H3K14ac), and histone H3 trimethylation (H3K9me3) were evaluated as markers for gene silencing, transcriptionally active chromatin, and constitutive heterochromatin, respectively.

Initially, mRNA expression levels and enzymatic activity of NAGLU and GALNS were measured. We confirmed the enzymatic activity defect in all evaluated fibroblasts, which agrees with previous reports using these cells. Interestingly, some cell lines also had reduced mRNA levels compared to healthy donor cells^29,34,35,52^. It is unlikely that the observed reductions in mRNA levels are explained by point mutations but rather suggest an additional mechanism regulating the levels of *NAGLU* and *GALNS* with a potential role in the pathophysiology of the diseases. Indeed, transcriptome studies have demonstrated that in cellular models of MPSs, the expression of genes coding for proteins involved in the regulation of the expression of many other genes at various stages is significantly altered^12,14,15^. This suggests that a more general mechanism leads to high differential dysregulation of gene expression control.

We also observed global DNA hypomethylation as a standard feature in both MPS IIIB and MPS IVA fibroblasts compared to healthy donor cells. A marked reduction in DNA methylation in the periphery of the nuclei and foci was also observed in MPS IIIB cells. DNA hypomethylation suggests a potential loss of epigenetic regulation in both diseases, which could be related to the altered gene expression patterns (signal transduction, transcription, splicing, RNA degradation, translation, and other genes) reported in fibroblasts from patients with MPS^12,14,15^. Altered gene expression patterns have also been reported during the progression of different human diseases with abnormal methylation levels (hypermethylation or hypomethylation) at CpG sites of crucial genes^53^. In the context of LSDs, altered DNA methylation for specific genes involved in the methylation pathway has been observed in a mouse model of Niemann-Pick Type C (NPC) disease^9^. Furthermore, previous studies have reported increased methylation levels in CpG sites associated with increased mutation probability in genes such as *GALNS*^5^ and *iduronate-2-sulfatase* (IDS)^6^. These findings highlight the importance of DNA methylation as a potential contributor to the phenotypic heterogeneity observed in LSDs.

Global hypomethylation is typical in aging cells and an early event in neoplasia linked to genetic instability^54,55^. Multiple studies have reported an association between altered DNA methylation and aneuploidy, increased chromosomal rearrangements, centromere instability, and consequent dysregulation of gene expression^56–58^. DNA damage associated with increased oxidative stress has been observed in MPS I, II, VI^59^, and MPS IVA cells, even after ERT treatment^60^. This highlights the need to consider the effect of treatments acting on secondary and tertiary impairments of the disease. Indeed, epigenetic changes have been associated with metabolic dysfunctions that could be particularly associated with specific phenotypic differences between patients with the same disease. Specifically, changes in some metabolites could affect the activity of the enzymes involved in DNA methylation and posttranscriptional modifications to histones^61^. Interestingly, global DNA hypomethylation has been linked to oxidative stress through the one-carbon cycle. Oxidative stress inhibits methionine synthase activity; this inhibition causes a reduction in the level of the methyl donor S-adenosylmethionine (SAM) while increasing the level of the methylation inhibitor S-adenosylhomocysteine (SAH). This altered SAM/SAH ratio reduces the availability of methyl groups, leading to DNA hypomethylation^62–65^. Altered metabolism associated with increased oxidative stress and its impact on DNA methylation through the one-carbon cycle highlights a potential role for nutritional intervention in MPS and other LSDs. Consumption of crucial nutrients such as folate (vitamin B-9), cobalamin (vitamin B-12), and methionine may promote methylation, which may have a future role in disease prevention and/or therapy^62^.

To have a broader perspective on epigenetic changes in MPS IIIB and IVA, we evaluated histone acetylation levels, specifically histone H3 lysine 14 acetylation (H3K14ac), an open chromatin marker. We observed that global and peripheral histone acetylation levels were increased in GM02931 MPS IIIB and GM00958 MPS IVA fibroblasts. Since H3K14ac is generally associated with transcriptional activation and open chromatin, it seems that in some patients, it may act synergically with DNA hypomethylation, inducing relaxed chromatin and potentially altering gene expression. Histone hyperacetylation and increased oxidative stress have been observed after excessive alcohol consumption and high-fat or high-glycemic diet impairing glucose and lipid metabolism^66,67^. Histone hyperacetylation is likely a cellular response to counteract sources of stress by enhancing the expression of genes involved in immunity and metabolism. Interestingly, bioactive compounds (BCs) such as carotenoids, bioactive fatty acids, peptides, polyphenols, glucosinolates, triterpene, and phytosterols are known to exhibit antioxidant, anti-inflammatory, and anti-cancer properties by regulating the cellular redox balance and histone acetylation state^66^. In this vein, it would be worthwhile to test the use of BCs as a potential approach to treating symptoms in MPS patients.

Finally, to gain insight into MPS IIIB and MPS IVA-related changes in the relatively stable heterochromatin, we tested the trimethylation of histone H3 in lysine 9 (H3K9me3), a constitutive heterochromatin marker. No significant changes in global or peripheral H3K9me3 levels were observed either in MPS IIIB or MPS IVA cells. The absence of global or peripheral alterations in H3K9me3 suggests that transcriptionally silent heterochromatin organization is not significantly impaired in MPS IIIB or MPS IVA H3K9me3. However, local reduction in H3K9me3 levels was observed in MPS IIIB fibroblasts, further pointing to abnormally open chromatin. We hypothesize that punctual foci H3K9me3 reduction could also result from diminished methionine levels induced by oxidative stress, as previously discussed for DNA hypomethylation.

Taken together, the analysis of all three markers points to complex changes in the epigenetic landscape in favor of abnormally open chromatin in MPS IIIB and IVA. The common trait for cells of both diseases was a reduction in DNA methylation. However, histone acetylation may also play a role in some MPS IIIB and IVA patients, while histone H3 trimethylation in lysine 9 may have a small impact on MPS IIIB patients. Besides punctual mutations' effect on enzyme activity and the specific genome context of each patient, the epigenetic changes identified in these cells may play a role in the phenotype exhibited by patients with the same disease.

The observed alterations in DNA methylation and histone acetylation are likely to influence the transcriptional activity of genes involved in the pathogenesis of the MPS. This is supported by reports showing widespread transcriptomic alterations in MPS patient fibroblasts^12,14,15^, hiPSC-derived chondrogenic cells with MPS VI^13^, and hippocampal cells of MPS VII mice^16^ This agrees with recent studies that have highlighted that MPS phenotype can be significantly influenced by secondary and tertiary biochemical and cellular changes related to numerous cellular processes within the cascade of changes in regulatory mechanisms rather than directly caused by GAG accumulation itself^12,14,15,17^. Furthermore, impaired gene expression might not be reversible, even after GAG level reduction using the isoflavone genistein^12,68^.

This study is in line with previous reports suggesting the involvement of epigenetic mechanisms in LSDs^2,3^. The observed alterations in DNA methylation and histone acetylation are consistent with studies conducted in other monogenic disorders^7,69,70^ and highlight the potential role of these epigenetic marks in modulating disease phenotypes. However, further investigation is required to uncover the precise molecular events responsible for the alterations in DNA methylation and histone acetylation observed in MPS IIIB and MPS IVA. Interestingly, it has been recently reported that despite heparan sulfate accumulated in all MPS III subtypes, there is a significant variation in the gene expression patterns among those subtypes^18^. It would be valuable to determine the functional consequences of these epigenetic changes on the expression of genes associated with the pathogenesis of LSDs and their impact on lysosomal function. Additionally, exploring the interplay between DNA methylation, histone modifications, and other epigenetic mechanisms, such as microRNAs, could provide a more comprehensive understanding of the epigenetic landscape in LSDs.

## Conclusion

This study provides evidence of altered epigenetic regulation in MPS IIIB and MPS IVA. The findings highlight the potential contribution of DNA methylation and histone acetylation to the phenotypic variability and disease manifestations observed in LSDs. Understanding the epigenetic mechanisms underlying LSDs could lead to the development of novel therapeutic strategies targeting these pathways and potentially alleviating the clinical outcomes for patients with these devastating disorders.

## Data Availability

Gene expression data have been deposited in Mendeley Data: doi: 10.17632/jwk7rg9s7f.1. Other data supporting this study's findings are available from the corresponding author upon reasonable request.
